# The Sensory & Social Neglect Index: A Novel Multidimensional Risk Tool for Metabolic and Cognitive Decline

**DOI:** 10.1093/geroni/igaf122.4031

**Published:** 2025-12-31

**Authors:** Arjun/Aarti Sahai, Ruizhi Huang, Jinmyoung Cho

**Affiliations:** Saint Louis University, Saint Louis, Missouri, United States; Saint Louis University, Saint Louis, Missouri, United States; Saint Louis University, Saint Louis, Missouri, United States

## Abstract

Older adults experience multisensory impairment and low social engagement; their combined impact on metabolic and cognitive decline is rarely examined. Our objective was to develop a Sensory/Social Neglect Index (SNI), integrating four domains and assessing its association with diabetes and cognitive decline in older adults. Using cross-sectional data and multinomial logistic regression, we examined the relationship between SNI levels and diabetes/cognitive decline (alone/combined), adjusting for demographic/health covariates. Data was extracted from the 2020 National Health Interview Survey. The SNI was created by summing 4 domains from NHIS variables: dental neglect, hearing impairment, vision impairment, and social neglect, yielding a cumulative level from 0-4. Respondents (4,573 U.S. adults aged ≥65) were categorized into neither condition, diabetes only, cognitive decline only, or both. SNI levels were right-skewed, with 36.1% of participants at level 0, 35.5% at level 1, 19.5% at level 2, 7.4% at level 3, and 1.5% at level 4. Higher SNI levels were more common in older adults, racial/ethnic minorities, and nonmetropolitan residents. The risk of cognitive decline only was significantly higher at SNI=3 (ARRR=3.65, 95% CI:1.33–10.01) and remained elevated at level 4 (ARRR=4.26, 95% CI:1.25–14.51). The risk of both conditions rose from level 2 (ARRR=18.10, 95% CI:2.07–157.93) to level 4 (ARRR=91.33, 95% CI:6.03–1382.52), though wide confidence intervals reflect small sample sizes. Final analyses will refine model estimates/explore category consolidation. Results suggest that sensory/social neglect is associated with cognitive decline and diabetes. Incorporating SNI-based screening into primary care could identify at-risk individuals and foster preventive care.

